# PYTAF: A Python Tool for Spatially Resampling Earth Observation Data

**DOI:** 10.1007/s12145-020-00461-w

**Published:** 2020-04-22

**Authors:** Guangyu Zhao, Muqun Yang, Yizhao Gao, Yizhe Zhan, H. Joe Lee, Larry Di Girolamo

**Affiliations:** 1 Department of Atmospheric Sciences, University of Illinois at Urbana-Champaign, Urbana, IL USA; 2 The HDF Group, Champaign, IL USA

**Keywords:** Resample, Python, Pytaf, Nearest Neighbor, Swath, Grid

## Abstract

Earth observation data have revolutionized Earth science and significantly enhanced the ability to forecast weather, climate and natural hazards. The storage format of the majority of Earth observation data can be classified into swath, grid or point structures. Earth science studies frequently involve resampling between swath, grid and point data when combining measurements from multiple instruments, which can provide more insights into geophysical processes than using any single instrument alone. As the amount of Earth observation data increases each day, the demand for a high computational efficient tool to resample and fuse Earth observation data has never been greater. We present a software tool, called pytaf, that resamples Earth observation data stored in swath, grid or point structures using a novel block indexing algorithm. This tool is specially designed to process large scale datasets. The core functions of pytaf were implemented in C with OpenMP to enable parallel computations in a shared memory environment. A user-friendly python interface was also built. The tool has been extensively tested on supercomputers and successfully used to resample the data from five instruments on the EOS-Terra platform at a mission-wide scale.

## Introduction

Earth observation data collected from thousands of satellites in space and numerous aircrafts have revolutionized Earth’s studies and significantly enhance the ability to forecast weather, climate and natural hazards. The products from Earth observation data contain measurements or derived properties of the surface and atmosphere for geographic positions or areas on the earth, which are generally described in a reference geographic coordinate system. A common choice of horizontal coordinates for Earth observation data is latitude and longitude conventionally defined in the World Geodetic System (WGS-84). Latitude and longitude may or may not be explicitly provided in Earth data products depending on their storage format, the majority of which belongs to one the three categories: swath, grid, and point, whose definitions are provided at https://newsroom.gsfc.nasa.gov/sdptoolkit/docs/HDF-EOS_UG.pdf. In brief, swath data are stored in a format resembling the standard scanning dimensions (i.e., along or across the ground track for satellites). Most satellite Level 1 and Level 2 products are swath data with measurements or derived properties stored for each instantaneous field-of-view (pixel) for each sensor. Latitude and longitude are provided for all or some of the pixels in swath data products. For example, most Level 1 and Level 2 products for the Moderate Resolution Imaging Spectroradiometer (MODIS) on the EOS Terra platform are swath data [See https://earthdata.nasa.gov/collaborate/open-data-services-and-software/data-information-policy/data-levels for the definition of data level]. Latitude and longitude are provided for 1 km resolution MODIS pixels as a standard product, but not for 250 m and 500 m pixels. Grid data are similar to swath data in the storage format, but grid data are stored at “regular” grids on certain spatial reference systems (SRS). Latitude and longitude do not need to be provided for grid data, because the grid already contains location information and latitude and longitude, if necessary, can be derived or calculated from the grid information and the SRS. For example, all of Level 1 and Level 2 products for the Multi-angle Imaging Spectro Radiometer (MISR) instrument also on Terra are on the Space Oblique Mercator (SOM) grid. To the best of our knowledge, all of the NASA Level 3 products are also grid data, since they are generated by aggregating Level-2 products into regularly spaced grid at a much course resolution (i.e., 0.5° latitude by 0.5° longitude) than Level 2 products. Point data are a series of data records taken at irregular time intervals and at scattered geographic locations. Latitude and longitude for individual points are normally provided for point data. Many aircraft data and some satellite products (e.g., the Level 2 footprint products for the Clouds and the Earth’s Radiant Energy System (CERES)) are point data.

Earth science studies frequently involve resampling between swath, grid and point data when combing measurements from multiple instruments, which can provide more insights into geophysical processes than using any single instrument alone (e.g., Diner et al. 2004, Harshvardhan et al. 2009, Liang and Sun 2015). In this paper, our examples focus on resampling swath data since many satellite products are stored in swath and the computational cost of resampling between swath data is high.

**Fig. 1:**
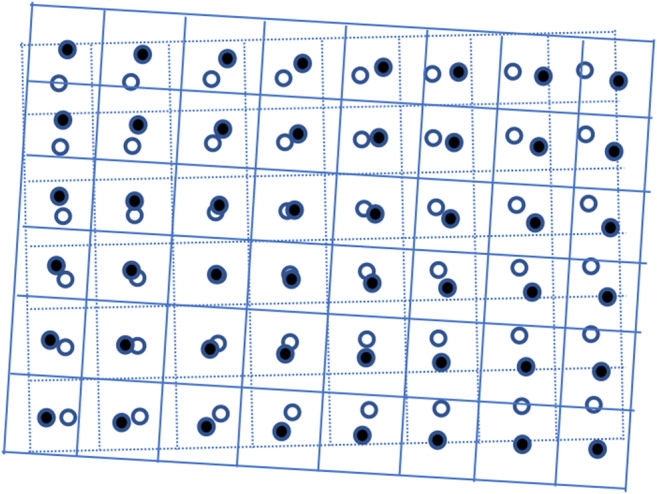
An illustration of resampling source swath data to target source swath data, represented by dotted and solid lines, respectively

An illustration of resampling between two swath data is given in Fig. 1, where squares surrounded by dotted and solid lines represent pixels of source and target swathes, respectively. The size of a square represents the resolution of a pixel. The centers of pixels are circled and their latitudes and longitudes are known. Resampling data from a source swath to a target swath is essentially to derive the values of the pixels in the target swath using the values of the pixels in the source swath. Resampling can be performed using nearest-neighbor (here after, NN), bilinear, or biquadratic interpolation algorithms, the choice of which relies on data type, application, and computational efficiency. Among these algorithms, NN is the fastest and simplest approach and it is applicable to swath, grid and point data. For each pixel in a target swath, the NN approach finds the NN pixel in the source swath that has the shortest spatial distance to it and assign the value of the NN pixel in the source swath to it. If the NN search is repeated for all of the pixels in a target swath, which is normally referred to as brute-force search or exhaustive search, the total number of operations will be the product of the total number of the pixels in the source swath and the total number of the pixels in the target swath. The time complexity of the brute-force searching algorithm is *O*(*MN*). For example, one Terra orbit (~98 min) has 188,467,200 MISR pixels at 275 m resolution and ~86,196,700 MODIS pixels at 250 m resolution, and resampling between MISR and MODIS using brute-force searching for one orbit would require 7.6 × 10^16^ operations. The computational cost dramatically increases with the size of datasets with the brute-force approach. A highly efficient algorithm is always in a great demand especially as the amount of Earth observation data increases each day.

In this paper, we present a software tool, called pytaf, which is built in C with a python interface to resample between swath, grid and point data using the NN approach. The tool was originally designed to resample data between all of the five instruments on EOS-Terra at a mission-wide scale with a total size of 1.3 Petabytes. The tool uses a novel block indexing algorithm to locate NN pixels. This searching algorithm significantly reduce the number of calculations of spatial distances. This algorithm is described in detail in section 2; Design and implementation are presented in section 3. Case studies are shown in section 4. The discussion and conclusion are given in section 5. Availability and requirements are provided in section 6.

## Block Indexing Algorithm Description

The block indexing algorithm was designed to refine and reduce the searching area for NN pixels. It would be physically meaningless to assign the value of the NN source pixel to a target pixel if the spatial distance between them is well beyond the spatial resolution of the target pixel. In other words, the NN searching in our context is bounded within a certain distance, *R*_*max*_, for each pixel in the target swath/grid/point. The block indexing algorithm can effectively locate all of source pixels that are within *R*_*max*_ to each target pixel.

**Fig. 2:**
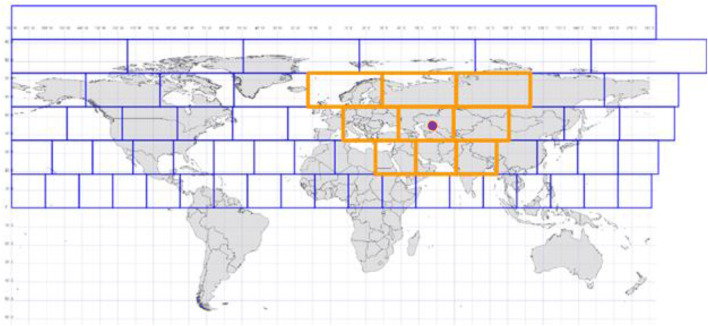
An illustration of block indexing

We divide the entire Earth surface into spatial blocks with an equal area of *R*_*max*_ by *R*_*max*_ in meters and create an integer index for each block. The latitude and longitude boundaries for each block are calculated in two steps. In the first step, the earth’s surface is split into horizontal strips from the north to south poles solely based on latitude. Each strip has a height of *R*_*max*_, corresponding to a latitude range of $180\times \frac{R_{max}}{R_{earth}}\times \pi$ in degree, where *R*_*earth*_ = 6,371.009 *km* (the Earth’s authalic (“equal area”) radius). In the second step, each strip is split into blocks from west to east with a width of *R*_*max*_. The longitude range for each block for each strip is $180\times \frac{R_{max}}{\max \Big(\ \cos \left( la{t}_{upper},\cos \left( la{t}_{lower}\right)\right)\times {R}_{earth}}\times \pi$ in degree, where *lat*_*upper*_ and *lat*_*lower*_ are the latitudes of the upper and the lower boundary of the strip. The number of blocks per strip decreases from the equator to the poles, where the strips closest to the poles have only one block each. An illustration of blocks is shown in Fig. 2.

These blocks are indexed with integer numbers and each source pixel is assigned an indexed number of the block it falls into by comparing its latitude and longitude with the latitude and longitude range of each block. Source pixels having the same index number belong to the same block. We can then identify the corresponding block that a target pixel falls into as well by comparing its latitude and longitude information with the latitude and longitude ranges of each block. The block that a target pixel falls into and its connected nearest neighboring blocks become the search area for the NN source pixels, and its NN within a distance of *R*_*max*_ must be in one of these blocks. As illustrated in Fig. 2, the red dot represents the location of a target pixel, and the nine original blocks defines the search area for the NN pixel. The maximum number of the candidate blocks that will be searched for NN source pixels is 9.

If no source pixels for a target pixel are found in the candidate blocks, a filled value of −999 will be assigned to the target pixel. Otherwise, the spherical great-circle distances between all of source pixels in the candidate blocks and a target pixel are calculated and the NN source pixel is identified as the one with the smallest distance. The great-circle distance is calculated using Eq. (1).

1 $$ Dis\left({s}_i,{t}_j\right)={R}_{earth}\ast arcc\mathrm{o}s\Big(\mathit{\sin}\left({lat}_{s_i}\right)\mathit{\sin}\left({lat}_{t_j}+\mathit{\cos}\left({lat}_{s_i}\right)\mathit{\cos}\left({t}_j\right)\mathit{\cos}\left({lon}_{s_i}-{lon}_{t_j}\right)\right) $$

where *R*_*earth*_ is the radius of the Earth, *s*_*i*_ and *t*_*j*_ represent the *i*th source pixel and *j*th target pixel, respectively, and *lat* and *lon* represent the latitude and longitude values in degree, respectively.

Note that if *R*_*max*_ is less than the spatial resolution of a source pixel for swath data, there should at most one source pixel in each of the candidate blocks. Hence, searching the NN source pixel for each target pixel only requires at most 9 spherical distance calculations. In this case, the time complexity of this algorithm is *O*(*N*), which is much smaller than the brute-force algorithm when *N* is much large than 9. In eq. (1), *R*_*earth*_ = 6,371.009 *km* with an assumption that the Earth is a sphere.

The tool, pytaf, will assign a value to the target pixel in two different ways depending on the difference in spatial resolution between target and source pixels. If a target pixel has a similar or much higher resolution than its NN source pixel, then its value will be equal to the value that the NN source pixel carries. For a scenario where the resolution of source pixels is much higher than that of target pixels, we reverse the NN searching between target and source datasets first. Instead of searching the NN source pixel for each target pixel, the NN target pixel is searched for each source pixel using the same block indexing searching algorithm. Then all of the source pixels that have the target pixel as the NN one will be aggregated and their mean value will be assigned to the target pixel. Besides their mean value, the standard deviation as well as the number of these source pixels is also reported for each target pixel. A good example for this scenario is given in Zhao and Di Girolamo (2006), where the ASTER 15 m data were resampled to the MISR grid at 1.1 km resolution.

## Design and implementation

The core NN searching functions of the pytaf tool were implemented in C. OpenMP was also used to enable parallel computations in a shared memory environment. Two python functions, resample_n and resample_s, were built to interface python to the C functions using Cython (Behnel et al. 2011). The code makes a large use of the Numpy library (Walt et al. 2011). The function of resample_n is used to resample data when target pixels have a similar or much higher resolution than their NN source pixels, while resample_s is used to resample data when source pixels are at a higher resolution than target pixels as the two scenarios discussed in the last paragraph in Section 2. Both functions of resample_n and resample_s have the same six input arguments as follows: 1) latitudes of all source pixels; 2) longitudes of all source pixels; 3) latitudes of all target pixels; 4) longitudes of all target pixels; 5) data values of all source pixels; 6) the maximum search radius, *R*_*max*_. For resample_n, *R*_*max*_ is normally chosen as the source pixel resolution, while *R*_*max*_ can be chosen as the target pixel resolution for resample_s.

The output of resample_n is a data array that contains resampled values at target swath/grid/point. The output data array has the same layout as the input latitude and longitude data arrays for target pixels do in memory. Besides this data array, resample_s has two additional output arrays. One reports the count of source pixels that contribute to each target pixel. The other reports the standard deviation of the values of these source pixels as discussed in the last paragraph of Section 2. The function of resample_s only counts source pixels with valid values great than zero for aggregation. For the function of resample_s, any pixel having a data value equal to or less than zero is not resampled. When no NN pixels are found or all of the NN pixels carry invalid values for a target pixel, a filled value of −999 will be assigned to the target pixel.

## Application

**Fig. 3:**
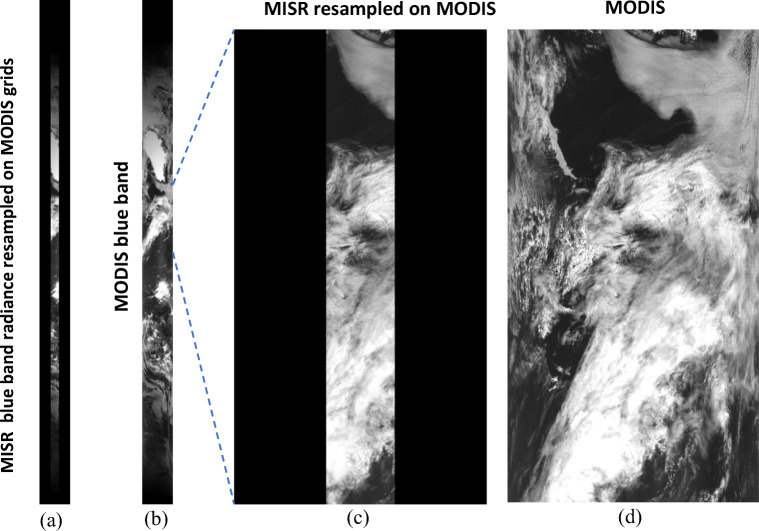
(a)The image of the MISR AN blue band radiance fields resampled on the MODIS grids for Orbit 71,826 taken on June 19, 2013, along with (b) the image of corresponding MODIS radiance fields for band 3. A subset region of the MISR image (a) and that of the MODIS image (b) are enlarged and displayed in (c) and (d), respectively

Figure 3 shows an example of the use of resample_n to resample the MISR AN blue band radiance data at 1.1 km resolution to the MODIS swath at 1.0 km resolution for the daylight side of a Terra orbit, which consists of 17 MODIS 5-min granules and one MISR granule. As shown in Fig. 3, the cloud features and patterns in the resampled MISR scene match the ones in the original MODIS scene perfectly. The total numbers of source (MISR) and target (MODIS) pixels are 46,726,540 and 8,257,536 respectively. The runtime for this case is 40 s using 32 threads, the maximum number of threads available on a single node, of the Blue Waters supercomputer at the National Center for Supercomputer Applications (NCSA) with OpenMP enabled.

**Fig. 4:**
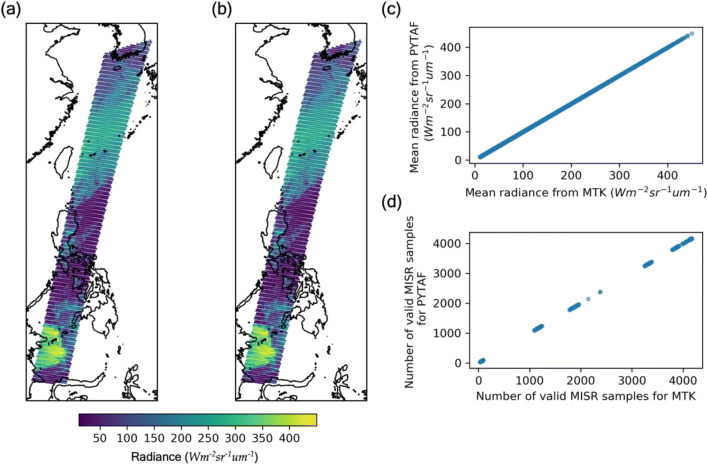
Comparison of MISR-CERES collocation between a benchmark search approach (MISR-Toolkit) and pytaf. (a) MISR 275-m red band samples were collected and averaged over 5000 CERES FOVs using MISR-Toolkit. (b) same as (a) but using pytaf. (c) A direct comparison of the calculated mean red band radiances between these two approaches. (d) same as (c) but for the number of valid MISR samples

To evaluate the efficiency of using pytaf to resample data with distinct spatial resolutions, Fig. 4 shows an example of resampling the MISR AN red band radiance data at 275 m resolution to the CERES near-nadir pixels (viewing zenith angle <20°) at 20 km resolution, which were part of a Terra orbit (orbit number 53557). The total numbers of source and targets pixels are 188,743,680 and 5000 respectively. By comparing the resample_s method to a brute-force method making use of the MISR-Toolkit (Zhan et al. 2018, Remote Sensing), we found that resample_s method provided robust results with much less runtime. By evenly separating the total 5000 target pixels into 3 subgroups, the total runtime of pytaf collocation is 23 s using 8 threads on a single node (Intel Xeon E5-2660v3) of the Keeling cluster at the School of Earth, Society, and Environment (SESE) at the University of Illinois while MISR-Toolkit-collocation took 5000 s to complete on the same machine. Among the total 4388 valid collocated MISR-CERES pixels, more than 90% samples (*n* = 3942) are identical between the two methods. The rest samples (*n* = 446) were found only to have 1~2 MISR pixel(s) different and resulted in a mean absolute difference of 0.009 Wm^−2^sr^−1^μm^−1^, which was equivalent to a mean relative difference of 0.009% of the MisrToolkit-collocation results. The 1~2 MISR pixel(s) difference is likely caused by difference in the spatial distance formula the two different methods use. The MISR-Toolkit uses the cartesian distance in calculating spatial distances, which is less accurate and less computationally expensive than the great circle formula that pytaf uses.

## Discussion and conclusion

We present a software tool, called pytaf. This tool resamples swath, grid or point Earth observation data into another swath, grid or point storage structure using nearest-neighbor search. The tool was specially designed to process large datasets such as mission-wide satellite products. In fact, it was initially developed to fuse the Terra datasets for the entire mission with a total size of 1.3 Petabyte as part of a NASA ACCESS funded program, named “ACCESS to Terra Data Fusion Product”, whose description is avaible at https://earthdata.nasa.gov/esds/competitive-programs/access/terra-data-fusion-products.

The tool has been extensively tested on the Blue Waters supercomputers. It has been used to project the MODIS level 1 radiance swath data onto the MISR grid, to resample ASTER radiance data into the MISR grid and MODIS swath, and resample MISR and MODIS radiance data into the CERES footprints at a large scale.

Although only nearest neighbor search is implemented in pytaf, it uses a novel and fast search algorithm, called block indexing. Bilinear and other weighting methods may yield more accurate results than nearest neighboring algorithm, but their computational cost is also much higher. Therefore, we did not implemented them in this tool. However, the open source nature and structure of this tool makes it easy for any developers/users to implement these features. The search algorithm was implemented in C with OpenMP for parallel computation with a user-friendly python interface. The search algorithm is computational efficient but it consumes more memory than conventional approach, because the block indices computed for each source and target pixels need to be stored in memory.

Great-circle distance instead of cartesian distance on the Earth are calculated in search of NN pixels for a high accuracy. The Earth is assumed to be a sphere rather than spheroid when calculating great-circle distance, which may lead to non-negligible round errors when the spatial resolution of the source dataset is at a scale of a few meters or below. The tool provides two resamples python functions: one resamples data where source pixels have a similar or lower spatial resolution than target pixels and the other resamples data where source pixels have much higher spatial resolution than target pixels.

The tool of pytaf assumes that the variation of the shapes of source and target pixels across the entire datasets is negligible. Pytaf uses a user-defined constant maximum searching radius for NN searching. We assume that the values that source pixels carry are uniformly distributed within them. In other words, subpixel variabilities are not considered during the resampling process.

## Availability and requirements

The pytaf tool is available for download at Github (https://github.com/TerraFusion/), along with the user guide, installation instructions, and the other tools for processing the Terra radiance data and fusion data. The tool was developed and tested with python 3.6.6 using Anaconda and pip on Microsoft windows 10, MacOS Mojave 10.14.5, Linux CentOS 7.2, and on NCSA Bluewaters, the cray XE/XK hybrid supercomputer. Unit test is automated by Travis-CI on GitHub. A simple example python script is also provided to help users with the usage of these two functions at the Github repository along with a user guide.
